# Triggering Germination Represents a Novel Strategy to Enhance Killing of *Clostridium difficile* Spores

**DOI:** 10.1371/journal.pone.0012285

**Published:** 2010-08-19

**Authors:** Michelle M. Nerandzic, Curtis J. Donskey

**Affiliations:** 1 Research Service, Cleveland Veterans Affairs Medical Center, Cleveland, Ohio, United States of America; 2 Geriatric Research, Education and Clinical Center, Cleveland Veterans Affairs Medical Center, Cleveland, Ohio, United States of America; Columbia University, United States of America

## Abstract

**Background:**

*Clostridium difficile* is an anaerobic, spore-forming bacterium that is the most common cause of healthcare-associated diarrhea in developed countries. Control of *C. difficile* is challenging because the spores are resistant to killing by alcohol-based hand hygiene products, antimicrobial soaps, and most disinfectants. Although initiation of germination has been shown to increase susceptibility of spores of other bacterial species to radiation and heat, it was not known if triggering of germination could be a useful strategy to increase susceptibility of *C. difficile* spores to radiation or other stressors.

**Principal Findings:**

Here, we demonstrated that exposure of dormant *C. difficile* spores to a germination solution containing amino acids, minerals, and taurocholic acid resulted in initiation of germination in room air. Germination of spores in room air resulted in significantly enhanced killing by ultraviolet-C (UV-C) radiation and heat. On surfaces in hospital rooms, application of germination solution resulted in enhanced eradication of spores by UV-C administered by an automated room decontamination device. Initiation of germination under anaerobic, but not aerobic, conditions resulted in increased susceptibility to killing by ethanol, suggesting that exposure to oxygen might prevent spores from progressing fully to outgrowth. Stimulation of germination also resulted in reduced survival of spores on surfaces in room air, possibly due to increased susceptibility to stressors such as oxygen and desiccation.

**Conclusions:**

Taken together, these data demonstrate that stimulation of germination could represent a novel method to enhance killing of spores by UV-C, and suggest the possible application of this strategy as a means to enhance killing by other agents.

## Introduction

*Clostridium difficile* is an anaerobic bacterium that is the most common cause of healthcare-associated diarrhea in developed countries [1]. During the past decade, the emergence of an epidemic *C. difficile* strain, termed North American pulsed-field gel electrophoresis type 1 (NAP1) or restriction endonuclease analysis (REA) type BI, has been associated with large outbreaks of *C. difficile* infection (CDI) in North America and Europe [1], [2]. These outbreaks have posed enormous challenges for infection control programs in hospitals and long-term care facilities. One feature of *C. difficile* that is particularly problematic for control efforts is the formation of spores. *C. difficile* spores are resistant to killing by alcohol-based hand hygiene products and by antimicrobial soaps that are commonly used in healthcare facilities [3]. *C. difficile* spores survive for months on surfaces and are resistant to killing by many commonly used disinfectants [4]. Moreover, low levels of some disinfectants may actually promote increased sporulation by *C. difficile* [5], [6]. Sodium hypochlorite (bleach) is a disinfectant with sporicidal activity, but it has several disadvantages, including being corrosive to many materials, irritating to some patients and staff members, and dependent on correct application by housekeepers [7]. There is a need for new strategies to reduce the burden of *C. difficile* spores on skin and in the environment.

Spore germination is defined as the irreversible loss of spore-specific properties and is an essential step required prior to outgrowth of vegetative cells [8], [9]. Because germinated spores become more susceptible to killing by heat and other stressors, induction of germination could be a potential strategy to facilitate eradication of *C. difficile* spores. This strategy has been studied as a possible measure to eliminate spores from food products (i.e., addition of germinants to reduce heat resistance of spores) [10]. Initiation of germination has been shown to increase susceptibility of spores of *Bacillus* spp. (*B. subtilis, B. coagulans*, and *B. cereus*) and *Clostridium botulinum* to killing by radiation and heat [11]–[15]. It is not known if initiation of germination of *C. difficile* spores results in similar increased susceptibility to killing by radiation or a variety of other stressors. However, Wheeldon et al. [16] recently demonstrated that exposure of *C. difficile* spores to the germinant sodium taurocholate resulted in increased susceptibility to killing by copper.

We previously demonstrated that a mobile, automated room disinfection device that utilizes ultraviolet-C (UV-C) radiation is effective in killing *C. difficile* spores and other pathogens (i.e. the Tru-D™ Rapid Room Disinfection device, Lumalier, Memphis, TN) (author's unpublished data). Although promising, the UV-C device did not completely eradicate spores from surfaces and ∼45 minutes were required to disinfect a 1-bed hospital room on the spore setting (i.e., a reflected dose of 22,000 µWs/cm^2^). Here, we tested the hypothesis that triggering germination could provide a novel strategy to enhance UV-C-induced killing of *C. difficile* spores on surfaces, thereby reducing the time and radiation dose necessary for disinfection of hospital rooms with the UV-C device. In addition, we evaluated the potential for initiation of germination to enhance killing of *C. difficile* spores by heat, alcohol, and exposure to room air.

## Materials and Methods

### Ethics Statement

Bacterial strains isolated from patients were collected from the Cleveland Veterans Affairs Medical Center. The Institutional Review Board of the Cleveland VA Medical Center approved the study protocol for collection of all patient isolates. Informed consent was not obtained because the isolates were cultured from clinical samples with no collection of patient identifiers or interaction with subjects.

### *C. difficile*, MRSA, and VRE Strains

Two *C. difficile* strains from the American Type Culture Collection (ATCC) and 2 strains cultured from patients with CDI in Cleveland were studied. ATCC 43593 and 43598 are from serogroups B and F, respectively. VA 17 is an epidemic restriction endonuclease analysis (REA) BI strain and VA 11 is an REA J strain. An MRSA pulsed-field gel electrophoresis type USA300 (i.e., community-associated MRSA strain) and a vanB-type VRE isolate (C68) were included for comparison in the initial UV-C experiments.

### Preparation of *C. difficile* Spores

Spores were prepared by growth on Duncan and Strong agar medium as previously described [17], [18]. Briefly, pre-reduced Duncan-Strong plates were spread with 100 µL of a four hour culture (0.8 McFarland) of *C. difficile* grown in enriched brucella broth. The plates were incubated for one week at 37°C in an anaerobic chamber and then for one week at room temperature on the bench-top. Spores were harvested from the plates using sterile swabs and two mL of sterile distilled water and absolute ethanol (50% final concentration). Spores were washed four times by centrifuging at 3000×g for 5 min and re-suspending in 1 mL of sterile distilled water. Spores were stored at 4°C in sterile water until use. Prior to testing, spore preps were confirmed by phase contrast microscopy and malachite green staining to be >99% dormant, bright-phase spores.

Spores were also prepared similarly on BHI agar and TSA supplemented with 5% sheep blood. There were no significant differences in the results of any of the experiments performed based upon the spore preparation medium (author's unpublished data).

### Germination *of C. difficile* Spores

Germination was induced using a defined medium consisting of amino acids, minerals and taurocholic acid as listed in Table 1. Germination was confirmed by two methods: 1) bright (dormant spores) to dark (germinating spores) phase transition under phase contrast microscopy and 2) a modified Wirtz-Conklin stain was performed as previously described by Hamouda et al. [19] to differentiate between dormant (green) or germinated (pink) spores.

**Table 1:** Formulation of *Clostridium difficile* spore germination solution consisting of amino acids, minerals and taurocholic acid prepared in sterile deionized water.

Component	Concentration	Component	Concentration
	(mg/L)		(mg/L)
Amino acid		Mineral	
Histidine	100	KH_2_PO_4_	300
Trytophan	100	Na_2_HPO_4_	1500
Glycine	100	NaCl	90
Tyrosine	100	CaCl_2_.2H_2_O	26
Arginine	200	MgCl_2_.6H_2_O	20
Phenylalanine	200	MnCl_2_.4H_2_O	10
Methionine	200	(NH_4_)_2_SO_4_	40
Threonine	200	FeSO_4_.7H_2_O	4
Alanine	200	CoCl_2_.6H_2_O	1
Lysine	300	NaHCO_3_	5000
Serine	300		
Valine	300	**Bile salt**	
Isoleucine	300	Taurocholic acid	1000
Aspartic acid	300		
Leucine	400		
Cysteine	500		
Proline	600		
Glutamic acid	900		

The amount of spores used, application of the germination medium and length of time spores were exposed to the germination medium were variable and described in detail for each individual experiment.

### Determination of Optimal Doses of UV-C for Killing of Germinating Versus Dormant *C. difficile* Spores, MRSA, and VRE

Initial experiments were conducted with spores of one strain of *C. difficile* (strain VA17) to determine if germination of spores in room air results in enhanced killing by UV-C and to determine the optimal UV-C doses for killing of spores on surfaces. We also examined optimal doses of UV-C for killing of MRSA and VRE. Ten µl aliquots of the organisms were allowed to air dry on laboratory bench tops 3 feet from the UV-C device. Surfaces inoculated with *C. difficile* spores were sprayed with either sterile deionized water (i.e., negative controls) or germination solution 5 minutes before UV-C was administered. The surfaces were subjected to specific reflected doses of UV-C radiation with the device or left untreated (i.e., controls). Pre-moistened swabs were applied to the surfaces and plated directly onto selective agar plates and the number of colonies recovered was counted. For *C. difficile* recovery, the swabs were transferred to an anaerobic chamber (Coy Laboratories, Grass Lake, MI) and plated onto pre-reduced *C. difficile* brucella agar (CDBA) [18]. Swabs from VRE and MRSA specimens were plated onto Enterococcosel agar (Becton Dickinson, Cockeysville, MD) containing 20 µg/mL of vancomycin and CHROMagar (Becton Dickinson) containing 6 µg/mL of cefoxitin, respectively. All plates were incubated for 48 hours.

For each pathogen, the inoculum applied to the bench top was adjusted such that 10^4^ to 10^5^ colony-forming units (CFU) were recovered from the control specimens. Reflected UV-C doses ranging from 5,000 to 22,000-µWs/cm^2^ were applied; the time to apply the doses ranged from ∼5 to 45 minutes. The reflected doses recommended by the manufacturer are 12,000 and 22,000 µWs/cm^2^ for killing of vegetative organisms and spores, respectively. The experiments were repeated four times.

### Examination of Susceptibility of Germinating and Dormant Spores to UV-C, Heat, and Ethanol

Because the initial experiments indicated that initiation of germination in room air increased susceptibility of spores of one *C. difficile* strain to UV-C, all 4 experimental strains were tested to determine the effect of initiation of germination on susceptibility to UV-C radiation at 12,000- µWs/cm^2^ (i.e., the vegetative killing dose). In addition, we assessed the effect of initiation of germination on susceptibility to heat (80°C for 5 minutes) and 90% ethanol. Ten µl aliquots of dormant spores (10^5^ CFU) were inoculated into 200 µl sterile deionized water or germination solution. Suspensions were mixed thoroughly to allow saturation with ambient oxygen and incubated at room temperature for 5 minutes. To assess susceptibility to UV-C radiation, 10 µl aliquots of each spore suspension were inoculated onto bench tops and left untreated (i.e., negative controls) or subjected to UV-C as previously described. Exposure to the dose of UV-C required ∼10 minutes. The spore counts on surfaces after exposure were assessed as described previously. Heat susceptibility was assessed by placing half of each spore suspension in an 80°C water bath for 5 minutes and ethanol susceptibility was assessed by adding 10 µl of each spore suspension to 90 µl of either absolute ethanol or sterile deionized water. After heat or ethanol exposure, the samples were transferred to the anaerobic chamber, serially diluted, and plated on CDBA agar. Following 48 hours of incubation, colonies of *C. difficile* were counted and log reduction was calculated. The experiments were repeated three times.

### Evaluation of UV-C Killing of Dormant and Germinating *C. difficile* Spores from Surfaces in Hospital Rooms

To evaluate the potential benefit of initiation of germination in a real-world setting, we assessed the effectiveness of UV-C radiation administered at a reflective dose of 12,000-µWs/cm^2^ (i.e., vegetative killing dose) for eradication of dormant versus germinating *C. difficile* spores from high-touch surfaces in hospital rooms. The call light, bedside table, telephone, bed rail and toilet were split into three areas and inoculated with spores of ATCC strain 43593 (∼10^4^ CFU in 10 µL aliquots). After drying in room air, the samples were sprayed with sterile deionized water (two areas) or germination solution (one area). One of the areas sprayed with water served as a before treatment control; pre-moistened swabs were used to collect samples before UV-C radiation. The remaining two areas were treated with UV-C radiation and pre-moistened swabs were used to collect post-treatment samples. The cultures for *C. difficile* were processed as described previously.

### Effect of Anaerobic Conditions and Increased Temperature on Ethanol Susceptibility of Dormant and Germinating Spores in Solution

Because initiation of germination did not increase susceptibility of spores to killing by ethanol in room air, we examined whether increased temperature and/or anaerobic conditions would facilitate killing of germinated spores by ethanol. Ten µl of dormant spores of epidemic strain VA17 (10^4^ CFU) were inoculated into 200 µl of oxygenated or pre-reduced germination solution. Aerobic and anaerobic spore suspensions were incubated at either room temperature (22°C) or in an incubator at 37°C. An anaerobic gas pack system (BD GasPak, Becton Dickinson, Franklin Lakes, NJ) was used to maintain anaerobic conditions outside of the anaerobic chamber. Ten µl aliquots were transferred into an anaerobic chamber at several time points during a 24 hour period and inoculated into 90 µl of absolute ethanol or sterile deionized water. The samples were serially diluted and plated on CDBA agar. Following 48 hours of incubation, colonies of *C. difficile* were counted and log reduction was calculated.

### Survival of Dormant and Germinating *C. difficile* Spores on Surfaces in Room Air

It is possible that initiation of germination might result in decreased survival of *C. difficile* spores on surfaces in room air by increasing susceptibility to desiccation, oxygen, or other stressors. Therefore, we assessed survival of dormant and germinating spores of the four *C. difficile* strains in room air either in solution or on surfaces. Ten µL of dormant spores were inoculated into 200 µL of either sterile deionized water or germination solution. Four aliquots (10 µL) of each spore suspension were inoculated onto four types of surface materials (i.e. plastic, wood, glass and laboratory bench top). Spore suspensions on surfaces dried within 30 minutes of inoculation. At several time points over a 24 hour period pre-moistened swabs were applied to the surfaces, transferred to the anaerobic chamber, and plated directly onto selective agar plates. Colonies recovered from the surfaces were counted at each time point and compared with baseline (time zero) levels of spores. Aliquots of the spores in either sterile deionized water or germination solution were also transferred into an anaerobic chamber at several time points during a 24 hour period and quantitative cultures were performed. Experiments were repeated twice.

### Data Analysis

Data were analyzed using STATA 9.0 (StataCorp, College Station, TX). Continuous data were analyzed using unpaired *t* tests and categorical data were assessed using Fisher's exact test.

## Results

### Determination of Optimal Doses of UV-C for Killing of Germinating Versus Dormant *C. difficile* Spores, MRSA, and VRE

**Figure 1:**
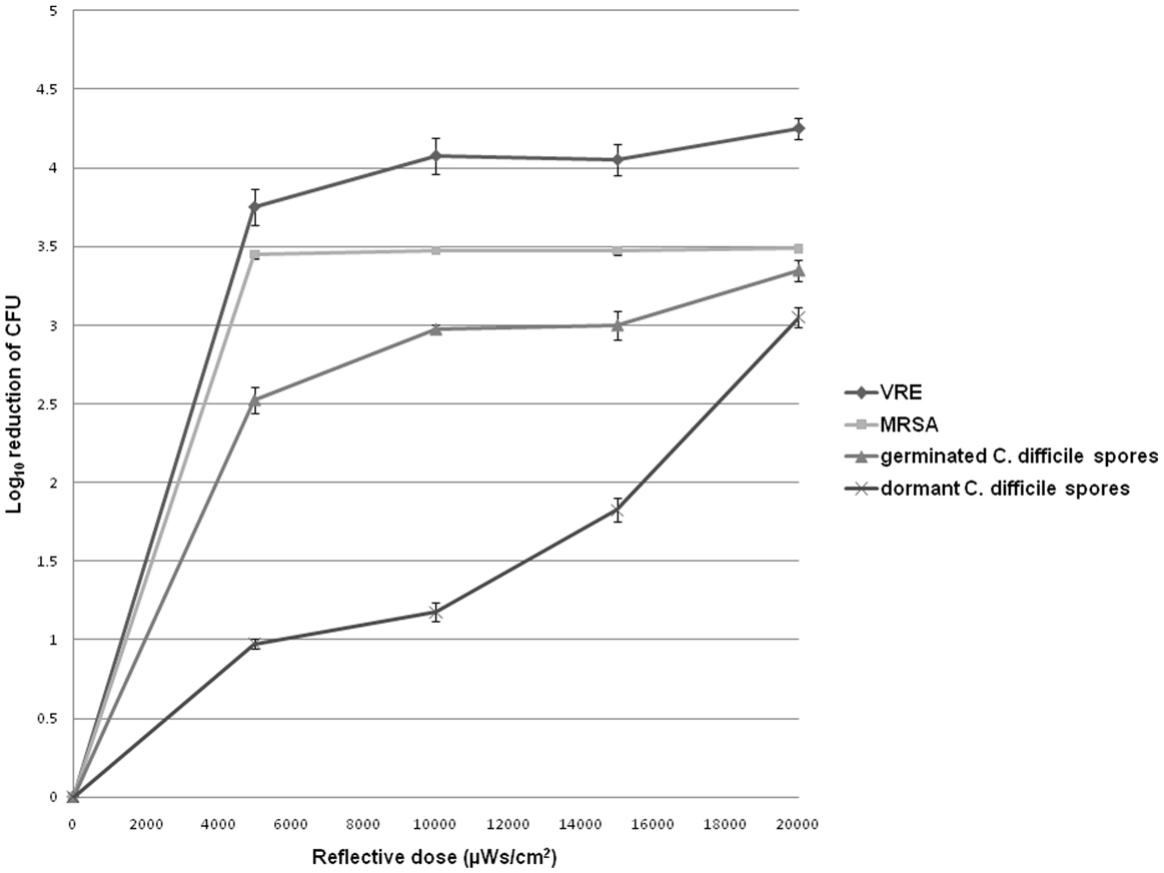
Optimal doses of ultraviolet-C radiation for killing germinating versus dormant *Clostridium difficile* spores, MRSA, and VRE. Mean reduction (log_10_ colony-forming units) in recovery of *C. difficile* spores from an epidemic NAP1/BI strain, a PFGE type USA 300 strain of methicillin-resistant *Staphylococcus aureus* (MRSA), and a vanB-type strain of vancomycin-resistant *Enterococcus* (VRE) from laboratory bench top surfaces after the use of a UV-C device at reflected doses ranging from 5,000 to 20,000 µWs/cm^2^. *C. difficile* spores were incubated in either water or germination solution for 5 minutes before exposure to UV-C radiation. Spores were confirmed as dormant or germinated by phase contrast microscopy.

Figure 1 shows the average reduction of *C. difficile* isolate VA17, with and without pre-treatment with germination solution, and of the MRSA and VRE test strains, in response to different reflected doses of UV-C. For the MRSA and VRE strains, greater than 3 log reductions occurred at each reflected dose with no enhancement of killing at higher doses. Killing of dormant *C. difficile* spores increased as the reflected dose increased, and a reflected dose of 20,000-µWs/cm^2^ requiring ∼45 minutes was required to achieve a 3 log reduction. In contrast, spores exposed to germination solution had increased susceptibility to UV-C such that a reflected dose of 10,000-µWs/cm^2^ requiring only ∼10 minutes was sufficient to achieve a 3 log reduction. It was confirmed that germination occurred within 5 minutes after exposure to the germination solution, as indicated by a change of spores from bright to dark phase under phase contrast microscopy and by change from green to pink color after staining with a modified Wirtz-Conklin stain [19].

### Examination of Susceptibility of Germinating and Dormant Spores to UV-C, Heat, and Ethanol

**Figure 2:**
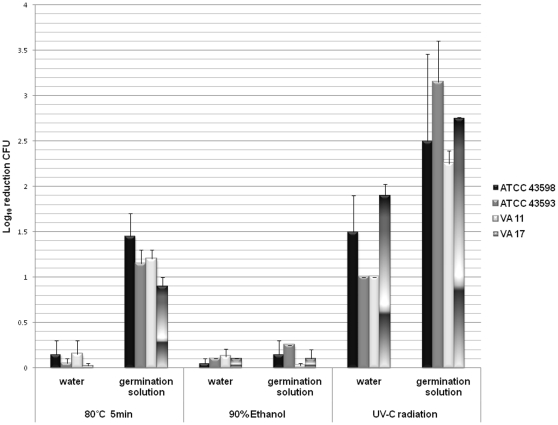
Examination of susceptibility of germinating and dormant spores to ultraviolet-C radiation, heat, and ethanol. Spores of the four experimental strains were incubated in either sterile deionized water or germination solution under ambient conditions for five minutes (confirmed as bright or dark-phase). Spores were exposed to ultraviolet-C (UV-C) radiation (12,000 µWs/cm^2^), heat (5 minutes at 80°C), or 90% ethanol. Mean reduction (log_10_ colony-forming units) in viable spores was assessed by enumerating serially diluted plate cultures before and after exposure to UV-C, heat and ethanol.

Figure 2 shows the mean reductions of dormant versus germinating spores in response to treatment with UV-C radiation at 12,000-µWs/cm^2^ (i.e., the vegetative killing dose), heat to 80°C for 5 minutes, and 90% ethanol. Dormant spores were not killed by heat or ethanol exposure, but UV-C treatment resulted in a 1 to 1.8 log reduction in dormant spores. Stimulation of germination did not enhance killing by ethanol (*P* = 1), but killing by heat and UV-C was enhanced (*P*<0.001 for germinated versus dormant spores). For UV-C exposure, the increase in killing for germinated versus dormant spores ranged from 0.85 log (strain VA17) to 2.15 log (strain ATCC 43593) (P<0.001 for each strain comparison).

### Evaluation of UV-C Killing of Dormant and Germinating *C. difficile* Spores from Surfaces in Hospital Rooms

UV-C treatment at a reflective dose of 12,000-µWs/cm^2^ for ∼20 minutes reduced recovery of dormant spores by 1.3 log (*P*<0.001). Stimulation of germination resulted in a further 0.9 log reduction (*P*<0.001). Germinating the spores reduced the frequency of positive cultures on commonly touched surfaces in patient rooms from 78% (44 of 52 sites) to 15% (6 of 27 sites) after a reflective dose of 12,000 µWs/cm^2^ (P<0.001).

### Effect of Anaerobic Conditions and Increased Temperature on Ethanol Susceptibility of Dormant and Germinating Spores in Solution

**Figure 3:**
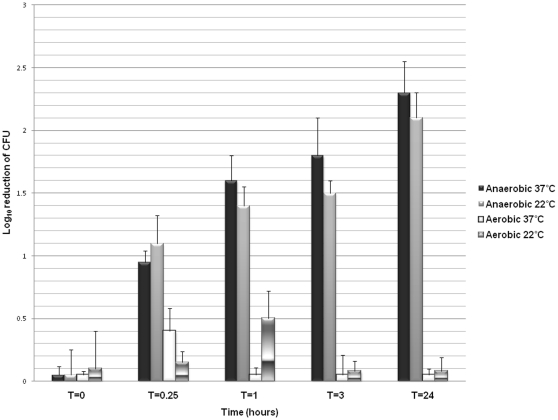
Effect of anaerobic conditions and increased temperature on ethanol susceptibility of dormant and germinating spores in solution. An epidemic NAP1/BI strain of *Clostridium difficile* spores was incubated in germination solution either aerobically or anaerobically at 22°C or 37°C. Mean reduction (log_10_ colony-forming units) in viable spores was assessed after exposure to 90% ethanol at several time points over 24 hours by enumerating serially diluted plate cultures.

Figure 3 shows the mean reductions (log_10_ CFU/mL) of spores germinating under various conditions after exposure to 90% ethanol. Germinating spores were not significantly reduced by exposure to 90% ethanol during the 24 hour testing period in the presence of room air at either 22°C or 37°C (*P* = 0.5). However, spores became increasingly susceptible to ethanol while germinating under anaerobic conditions for 24 hours. The increased susceptibility of spores to killing by ethanol was independent of temperature; there was no significant difference whether incubated anaerobically at 22°C or 37°C (*P* = 0.66). After 24 hours of exposure to germination solution under anaerobic condition, spores were dark phase and swollen under phase contrast microscopy however no mature vegetative cells were visualized.

### Survival of Dormant and Germinating *C. difficile* Spores on Surfaces in Room Air

**Figure 4:**
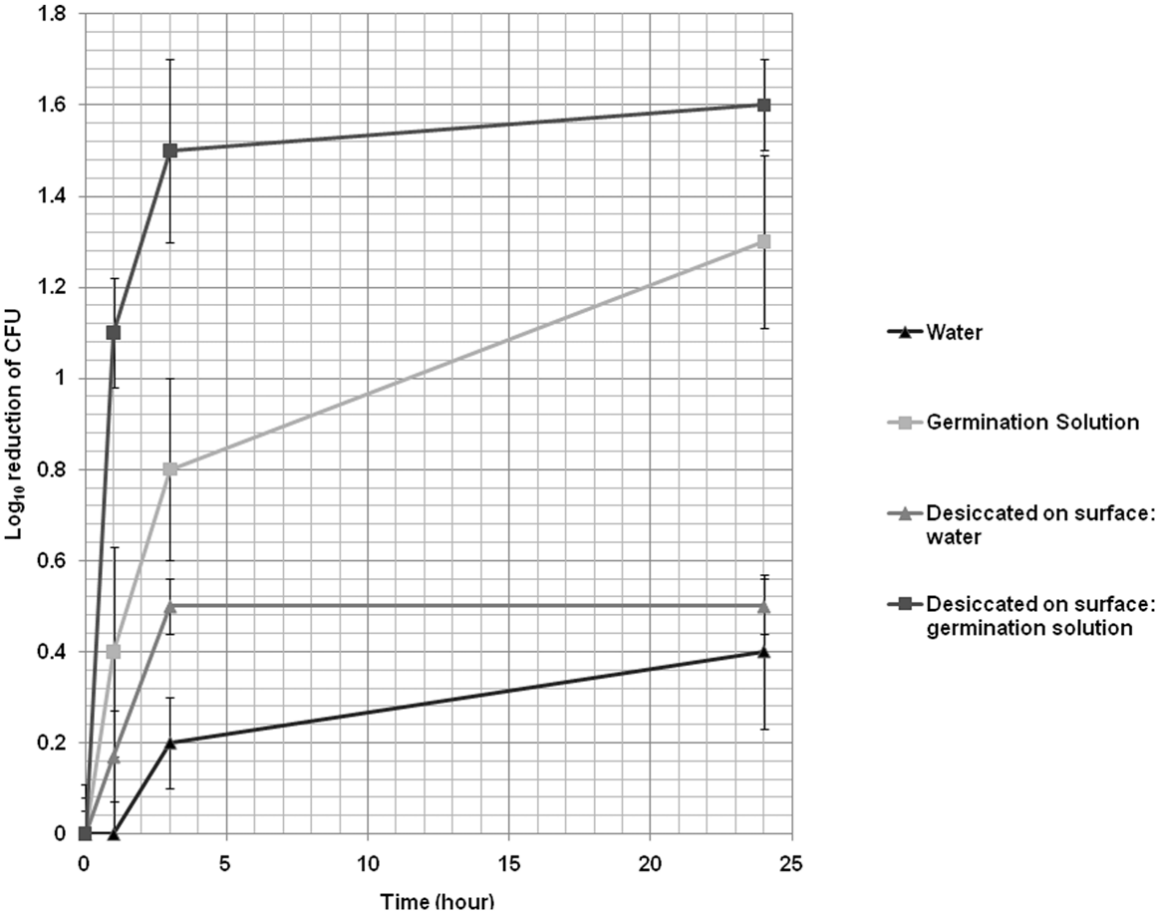
Survival of dormant and germinating *C. difficile* spores on surfaces in room air. Spores of the four experimental strains were incubated in sterile deionized water or germination solution under ambient conditions. Spore either remained in solution or were allowed to desiccate on surfaces over a 24 hour period. Mean reduction in recovery of viable dormant and germinating spores exposed to room air on surfaces (desiccated) was compared with baseline levels of spores inoculated onto surfaces. Mean reduction of viable spores in solution was assessed by enumerating serially diluted plate cultures at each time point in comparison with baseline levels of spores.

Figure 4 shows the mean reductions of dormant and germinating spores exposed to room air on surfaces (desiccated) or in solution. There was no significant difference among the four strains of *C. difficile* tested or in recovery of spores from different types of surfaces (i.e. plastic, wood, glass or laboratory bench top). Spores suspended in water were confirmed to be dormant by phase contrast microscopy during the 24 hour test period. In comparison to spores suspended in water or sprayed with water and desiccated on a surface, spores suspended in germination solution demonstrated significant reductions in viable counts recovered at 1, 3, and 24 hours (*P*<0.005). In comparison to spores suspended in germination solution, spores sprayed with germination solution and desiccated on a surface demonstrated a trend toward significant reductions in viable counts recovered at 1 and 3 (*P* = 0.07), but not 24 hours (*P* = 0.76).

## Discussion

We found that exposure of dormant *C. difficile* spores to germination solution resulted in initiation of germination in room air. Germination of the spores in room air significantly enhanced killing by UV-C radiation and heat, but not by ethanol. On surfaces in hospital rooms, application of germination solution resulted in significantly enhanced eradication of spores by UV-C such that the ∼15 minute vegetative cycle of the UV-C device provided a greater than 2 log reduction in recovery of spores. Stimulation of germination also resulted in reduced survival of spores on surfaces in room air, possibly due to increased susceptibility to stressors such as oxygen and desiccation. These findings demonstrate that stimulation of germination could represent a novel method to enhance killing of *C. difficile* spores by UV-C, and suggest the possible application of this strategy as a means to enhance killing by other agents.

Spore germination consists of two distinct constitutive stages [20]. Stage I is composed of three processes: 1) release of H^+^, monovalent cations and Zn^2+^ from the spore core (increasing core pH from ∼6.5 to 7.7), 2) release of dipicolonic acid and Ca^2+^ from the spore core, and 3) increase in the spore core's hydration. Stage II is composed of two processes: 1) hydrolysis of the spore's cortex, and 2) swelling of the spore core due to further increase in hydration and expansion of the germ cell wall. Following stage I and II of germination the conditions are favorable for metabolism to resume during outgrowth. Because vegetative cells are susceptible to killing by ethanol, our findings suggest that spore germination was initiated in room air by exposure to the germinant solution, but that the process was stopped at an intermediate point prior to outgrowth (i.e., germination of spores in room air did not result in susceptibility to killing by ethanol). In contrast, initiation of germination under anaerobic conditions did result in susceptibility to killing by ethanol, suggesting that exposure to oxygen might prevent *C. difficile* spores from progressing fully to outgrowth.

Our findings have important practical applications. First, reducing the time required for use of UV-C devices might significantly increase the feasibility of their use in hospital settings that require rapid disinfection of rooms. Our findings suggest that application of a germinant solution could reduce the time required for disinfection of CDI rooms from ∼45 minutes (i.e., the spore cycle) to ∼15 minutes (i.e., the vegetative cycle). Because spore contamination is not uncommon in non-CDI rooms in hospitals [21], [22], application of a germination solution in non-CDI rooms may be beneficial in outbreak situations. Second, stimulation of germination of *C. difficile* spores resulted in reduced survival on surfaces in room air. Therefore, stimulation of germination could potentially be beneficial as a strategy to reduce the burden of spores in the environment even in the absence of UV-C treatment. Finally, our findings should stimulate additional research to identify other stressors that might kill germinated spores. As noted previously, Wheeldon et al [16] recently reported that exposure of *C. difficile* spores to a germinant (sodium taurocholate) under aerobic conditions resulted in enhanced killing on copper surfaces.

Our study has some limitations. First, only 4 strains of *C. difficile* were studied. However, the results were consistent for each of the 4 strains. Second, it is not known which components of the germination solution are essential to stimulate germination to a stage in which enhanced killing by stressors such as UV-C are possible. Further studies of *C. difficile* germination are needed. Third, the studies in hospital rooms involved inoculation of spores onto surfaces rather than spore contamination from CDI patients. Additional studies are needed in CDI rooms. Fourth, it is possible that factors such as organic matter might reduce the efficacy of germination solutions and of UV-C. A previous study has demonstrated that gross particulate matter (silica powder) significantly reduced UV-C's effectiveness in killing spores [23]. However, in practice, hospital rooms would be cleaned to remove organic material prior to application of germination solution and UV-C. Finally, as previously described by Gould et al. [12] a persistant “superdormant” fraction of spores remains unaltered by exposure to germinants. More recently Ghosh and Setlow [24] isolated superdormant spores of *Bacillus subtilis* and *Bacillus megaterium* and found that super-dormant spores require an increased signal for triggering spore germination compared to most spores in populations. Further studies are necessary to optimize a germination solution for *C. difficile* that can further enhance killing by triggering germination in the super-dormant fraction of spores.
